# Detection and Isolation of Emetic *Bacillus cereus* Toxin Cereulide by Reversed Phase Chromatography

**DOI:** 10.3390/toxins13020115

**Published:** 2021-02-04

**Authors:** Eva Maria Kalbhenn, Tobias Bauer, Timo D. Stark, Mandy Knüpfer, Gregor Grass, Monika Ehling-Schulz

**Affiliations:** 1 Functional Microbiology, Institute of Microbiology, Department of Pathobiology, University of Veterinary Medicine Vienna, 1210 Vienna, Austria; evamaria.kalbhenn@vetmeduni.ac.at (E.M.K.); tobias.bauer@mein.gmx (T.B.); 2 Chair of Food Chemistry and Molecular Sensory Science, Technical University of Munich, Lise-Meitner-Straße 34, 85354 Freising, Germany; timo.stark@tum.de; 3 Bundeswehr Institute of Microbiology, Neuherbergstraße 11, 80937 Munich, Germany; mandyknuepfer@bundeswehr.org (M.K.); gregorgrass@bundeswehr.org (G.G.)

**Keywords:** cereulide, reversed phase chromatography (RPC), Äkta™ pure, peptide quantification, emetic *Bacillus cereus*, toxin purification

## Abstract

The emetic toxin cereulide is a 1.2 kDa dodecadepsipeptide produced by the food pathogen *Bacillus cereus*. As cereulide poses a serious health risk to humans, sensitive and specific detection, as well as toxin purification and quantification, methods are of utmost importance. Recently, a stable isotope dilution assay tandem mass spectrometry (SIDA–MS/MS)-based method has been described, and an method for the quantitation of cereulide in foods was established by the International Organization for Standardization (ISO). However, although this SIDA–MS/MS method is highly accurate, the sophisticated high-end MS equipment required for such measurements limits the method’s suitability for microbiological and molecular research. Thus, we aimed to develop a method for cereulide toxin detection and isolation using equipment commonly available in microbiological and biochemical research laboratories. Reproducible detection and relative quantification of cereulide was achieved, employing reversed phase chromatography (RPC). Chromatographic signals were cross validated by ultraperformance liquid chromatography–mass spectrometry (UPLC–MS/MS). The specificity of the RPC method was tested using a test panel of strains that included non-emetic representatives of the *B. cereus* group, emetic *B. cereus* strains, and cereulide-deficient isogenic mutants. In summary, the new method represents a robust, economical, and easily accessible research tool that complements existing diagnostics for the detection and quantification of cereulide.

## 1. Introduction

The emetic toxin, cereulide, is a dodecadepsipeptide, composed of six α-amino acid, and six α-hydroxy acid, moieties arranged in three repeating tetradepsipeptide units [D-O-Leu-D-Ala-L-O-Val-L-Val]_3_ [1]. Cereulide is produced by an emetic subgroup of *Bacillus cereus*, a bacterial pathogen typically associated with food poisoning [2,3]. Similarly to other highly bioactive peptides, such as the antibiotic valinomycin produced by *Streptomyces* spp., cereulide is synthesized by a non-ribosomal peptide synthetase (NRPS), designated CesNRPS [4,5,6]. The *cesNRPS* genes are organized as an operon within a 24 kb multigene cluster located on the pCER270 megaplasmid, which shares its backbone with the pXO1 toxin plasmid of *Bacillus anthracis* [7]. The structural *cesAB* genes, which play a pivotal role in cereulide biosynthesis, are co-transcribed as a single polycistronic mRNA with adjacent genes [8,9,10]. Cereulide accumulates during growth of emetic *B. cereus* in a growth temperature range from 12 °C to 40 °C [11,12], and reaches high levels in the stationary phase [8,13,14]. However, cereulide production capability among emetic *B. cereus* strains varies up to 1000-fold [4,15,16].

Due to its cyclic structure, cereulide is extremely resistant against heat, extreme pH conditions, as well as proteolysis [17], and cannot be inactivated by standard hygienic measures in food production and processing. Furthermore, cereulide is not inactivated during stomach passage in the host, because of the peptide’s resistance to cleavage by pepsin and trypsin [18,19,20]. Thus, cereulide represents a serious challenge for the food industry, as severe intoxications linked to cereulide are on the rise [12,21]. Key symptoms of cereulide intoxication are fulminant episodes of vomiting shortly after the consumption of cereulide contaminated food, nausea, and abdominal cramps [14]. Due to its hepatotoxic activity, cereulide can cause liver damage, rhabdomyolysis, and severe multi-organ failure [22,23,24,25]. Documented biological activities of cereulide include emesis in primates [18,26], and swelling of mitochondria in HEp-2-cells [27]. Cereulide has also been linked to the induction of diabetes by causing beta cell dysfunctions [28]. In addition, neurological symptoms, such as seizures and lethargy, similar to those described from human intoxications have been reported in intoxication studies using a porcine model [29]. The intoxication studies also showed that cereulide can accumulate in several organs and tissues, and possibly cross the blood–brain barrier [29].

Due to the lack of fast and specific cereulide quantification and isolation methods, purification and quantification of the toxin is still laborious. Nevertheless, considerable progress has been made in the diagnostics of emetic *B. cereus* [30], and methods for specific detection of emetic *B. cereus* strains by matrix-assisted laser desorption/ionization–time-of-flight (MALDI-ToF) mass spectrometry (MS) have been published recently [31,32]. Since MALDI-ToF MS is increasingly used in routine microbiology laboratories, it is expected that these methods, allowing the discrimination of emetic and non-emetic *B. cereus,* will significantly improve differential *B. cereus* diagnostics in clinical settings, as well as in foodborne outbreak situations. A drawback of the current MALDI-ToF MS methods are the mass spectrometers commonly used in routine diagnostics; which do not allow accurate quantification of cereulide [31]. Thus, liquid chromatography coupled to mass spectrometry (LC-MS) is still considered the gold standard for cereulide detection and quantification [33,34,35,36]. Based on stable isotope mass spectrometry-based dilution assay (MS-SIDA) [33], an EN-ISO method (EN ISO 18465) for quantitation of cereulide in food matrices was recently established [37].

However, although the latter MS method is characterized by high accuracy, its use in microbiological research is limited by the financial burden of the MS equipment required. We therefore aimed to develop a method suitable for research in microbiological and biochemical laboratories, using equipment commonly available there, such as reversed phase chromatography (RPC) systems for the purification of proteins, peptides, and nucleic acids. These flexible chromatography systems allow for quick, simple, and easy customization. Owing to their broad applicability and the low costs (compared to sophisticated UPLC-MS/MS), RPC systems are frequently available in microbiological and biochemical laboratories. Here, we employed an Äkta™ pure RPC chromatography system, to assess its suitability for cereulide research. Our work suggests that RPC systems are indeed valuable tools for cereulide toxin research, allowing, not only its detection, but also the purification, isolation, and relative quantification of cereulide.

## 2. Results and Discussion

### 2.1. Establishing a Work Flow for Cereulide Detection from Emetic B. cereus by RPC: Cultivation of Bacteria and Crude Cereulide Extraction (Step 1–2)

A set of strains for cereulide production, detection, and purification by RPC was compiled, including emetic *B. cereus* strains with different cereulide biosynthesis capacities, isogenic mutant strains, as well as non-emetic representatives of the *B. cereus* group. A list of strains included in this panel is provided in Table 1.

**Figure 1:**
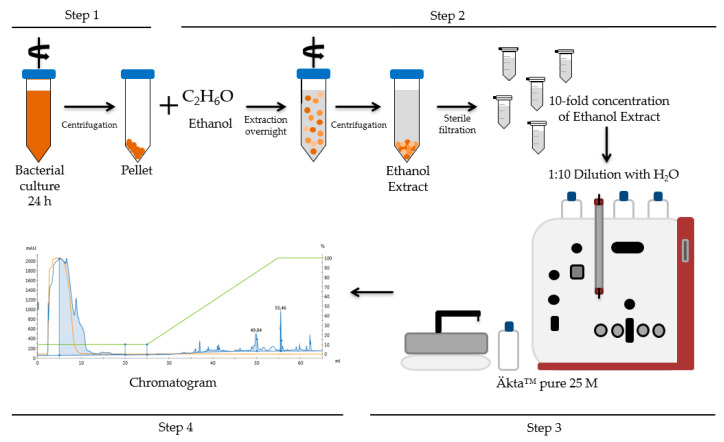
Schematic principle of cereulide analysis by reversed phase chromatography (RPC). Step 1: Cultivation of bacterial strains; Step 2: Extraction procedure; Step 3 + 4: Cereulide toxin isolation and quantification by RPC using an Äkta™ pure system and a silica based C12 column.

Strains were grown under standardized laboratory conditions in LB medium (Lysogeny broth medium) at 30 °C and rotary shaking at 120 rpm for 24 h, using a previously established protocol for kinetic inoculation to ensure reproducibility [5]. Cells were harvested by centrifugation, and subsequently cereulide was extracted from the cells at room temperature with ethanol overnight. Next, cells were pelleted by centrifugation and supernatants were collected. After removal of cell debris from the supernatants by filtration (0.2 µm filter size), the crude cereulide extract was either subjected directly to UPLC-MS/MS for quantitation (see Section 2.3), or the cereulide extract was processed for analysis and further cereulide isolation by RPC (see Section 2.2). A schematic overview of the complete workflow is depicted in Figure 1.

### 2.2. Identification and Isolation of Purified Cereulide Toxin (Step 3-4): Cereulide Chromatogram on an Äkta™ Pure 25M Using a Silica Based RP C12 Column

**Figure 2:**
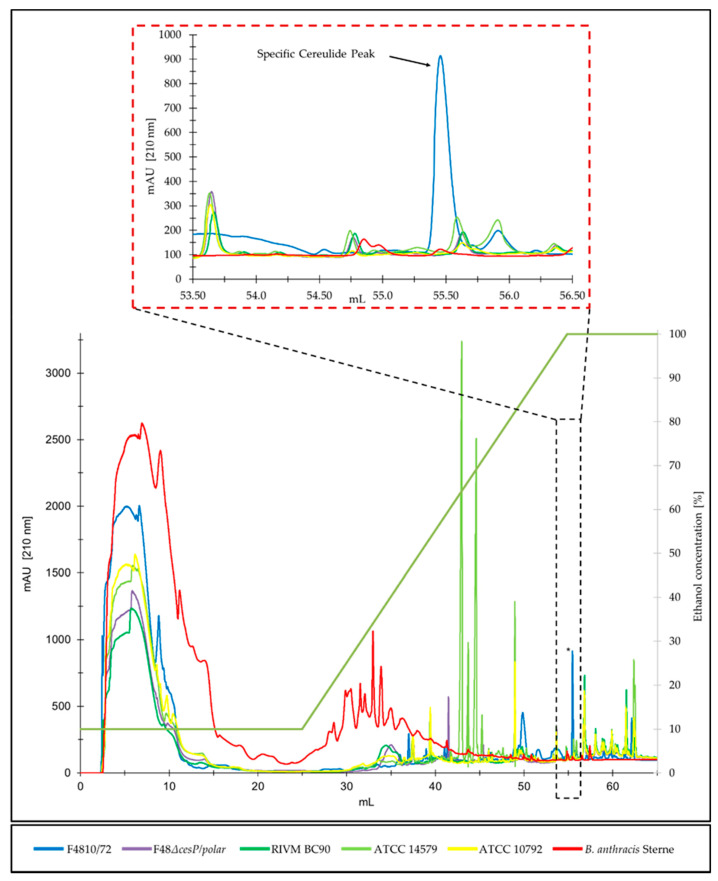
Reversed phase chromatogram of ethanol extracts from the emetic reference strain F4810/72 (blue) and the cereulide negative isogenic mutant F48*∆cesP/polar*^.^ (purple), as well as of selected non-cereulide producing *B. cereus* group strains: emetic-like *B. cereus* RIVM B90 (green), *B. cereus* type strain ATCC 14579 (light green), *B. thuringiensis* type strain ATCC 10792 (yellow), and *B. anthracis* Sterne (red). Strains were grown in LB (Lysogeny broth) for 24 h at 30 °C, cereulide was extracted from cells with ethanol absolute over-night, and concentrated 10-fold. Ethanol extracts were diluted 1:10 in water (*v*/*v*) and analyzed by RPC, as described in the material and method section. The specific cereulide peak at 55.5 mL (see inset) was collected using automatic peak sampling, and subsequently quantified by ultraperformance liquid chromatography–mass spectrometry (UPLC-MS/MS). A representative result from three independent experiments is shown.

Due to the highly hydrophobic character of cereulide [42], RPC was performed using a silica based C12 column and an Äkta™ pure 25 M instrument to separate hydrophobic substances from ethanol extracts of *B. cereus* group strains, and to identify cereulide. For separation of the metabolites in the ethanol extracts a linear gradient from 10% ethanol to ethanol absolute for 60 min, followed by 15 min at ethanol absolute was used. UV absorption at 280 nm (proteins) and 210 nm (peptides) was simultaneously recorded. A specific chromatographic signal during RPC at 210 nm after 55.5 mL was detected in the emetic reference strain F4810/72, while this peak was absent in all cereulide negative *B. cereus* group strains, such as the *B. cereus* type strain, the *Bacillus thuringiensis* type strain, and *Bacillus anthracis* (Figure 2). The fraction corresponding to the specific peak at 55.5 mL in the emetic reference strain F4810/72 was subjected to UPLC–MS/MS analysis and identified as cereulide (for details see Section 2.3).

Furthermore, this cereulide specific chromatographic signal was absent in the emetic like strain, RIVM BC90. Emetic like strains are strains that are genetically closely related to emetic strains, and share certain phenotypic characteristics with the latter, except that they do not possess *ces* genes and are thus unable to produce cereulide [2,3]. Based on the close relatedness, it could be expected that the metabolite pattern in the ethanol extracts of emetic like strains might be similar to the ones of emetic strains, except for the absence of cereulide. Similarly, it could be expected that the isogenic cereulide-deficient mutant F48*∆cesP/polar* of the emetic reference strain F4810/72 would share, excluding its cereulide deficiency, general phenotypic features with its parental strain, reflected in the ethanol extracts of metabolites. As shown in Figure 2, the metabolite pattern in the ethanol extracts of emetic-like strain RIVM BC90 and the cereulide-deficient mutant, F48*∆cesP/polar* were indeed more similar to the pattern of the emetic strain F4810/72, than to those of the more distantly related non-emetic *B. cereus* group strains. Yet, similarly to the chromatograms from the non-emetic *B. cereus* group strains, the specific peak detected in F4810/72 was absent from their respective chromatograms, fostering the hypothesis that the peak at 55.5 mL is specific to cereulide producing emetic *B. cereus*.

**Figure 3:**
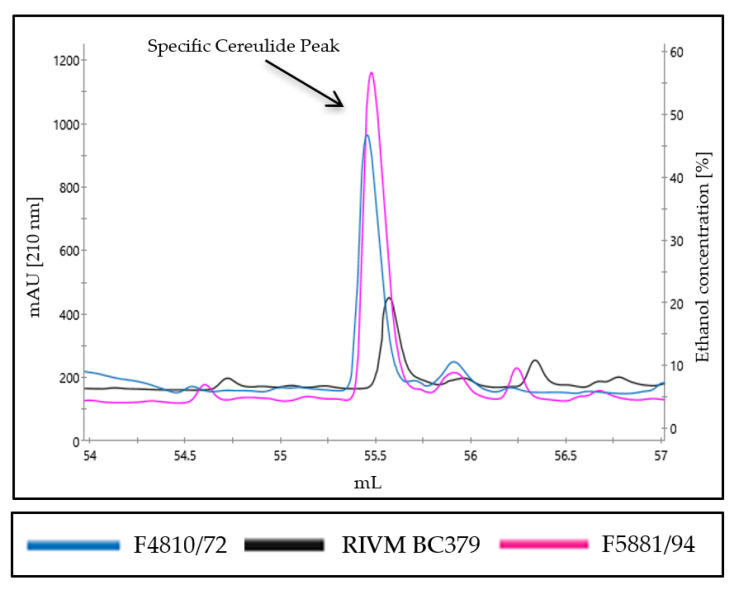
Reversed phase chromatogram of ethanol extract from emetic *B. cereus* strains with different cereulide production capacities, such as the medium toxin producer F4810/72 (blue), the high toxin producer F5881/94 (pink), and the low toxin producer RIVM BC379 (black). Strains were grown in LB broth, processed, and analyzed by RPC, as described in the material and method section. A representative result from three independent experiments is shown.

To test this hypothesis, we next analyzed two additional emetic *B. cereus* strains, one high (F5881/94), and one low, cereulide producer (RIVM BC379) [34]. As depicted in Figure 3, extracts of both strains showed the prominent peak at 55.5 mL, highlighting the specificity of this peak. Notably, the high cereulide producer strain F5881/94 exhibited a higher peak than the medium toxin producer strain, F4801/72, while the peak of the low cereulide producer (RIVM BC379) was even lower than the peak of F4801/72, indicating that our method may be used for relative quantification and classification of emetic strains (see Table 2).

**Figure 4:**
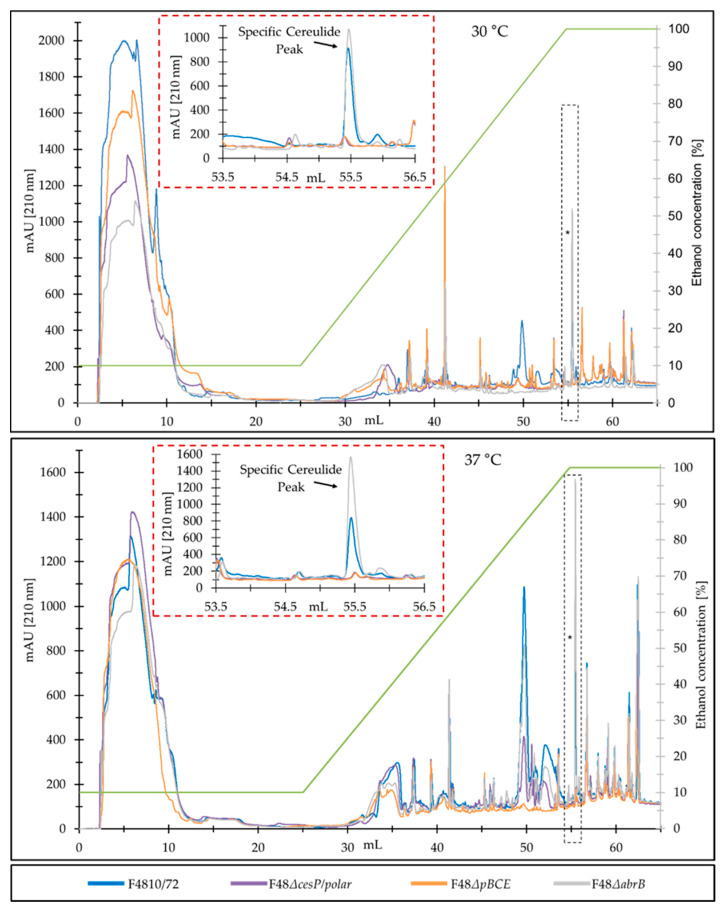
Reversed phase chromatogram of ethanol extracts from wildtype F4810/72 and its isogenic mutants. The specific cereulide peak at 55.5 mL from wildtype F4810/72 is indicated by an asterisk. The strains were grown in LB broth for 24 h at 30 °C or 37 °C, respectively, extracted with ethanol absolute over-night and concentrated 10-fold. Ethanol extracts were diluted 1:10 in water (v/v) and analyzed by RPC. The specific cereulide peak at 55.5 mL was collected and quantified by UPLC–MS/MS. A representative result from three independent experiments is shown.

Next, we tested the suitability of our new RPC method for a rapid analysis of the cereulide production capacities of mutant strains, as this allowed for a relative quantification of cereulide. To this end, two cereulide deficient mutant strains, one cereulide overproducing mutant and the parental strain F4810/72 were grown in LB broth, with rotary shaking at 120 rpm, for 24 h, at 30 °C and at 37 °C. Samples were processed as described above. As expected, RPC of the respective ethanol extract from the mutant strains revealed that the peak at 55.5 mL was absent in the cereulide-deficient mutants F48*∆cesP/polar* and F48*∆pBCE*, while it was higher in the cereulide-overproducing mutant F48*∆abrB* compared to the parental F4810/72 (Figure 4), reflecting the previously reported upregulated cereulide production in this mutant strain [40].

As shown in Figure 4, cereulide production in the *abrB* deletion mutant was increased even more at 37 °C than at 30 °C compared to the parental strain. The temperature effect on cereulide production in the F48*∆abrB* detected by our RPC method warrants further investigation, as AbrB is an important, pleiotropic transition phase regulator in Bacilli, which is still far from being fully understood.

The respective RPC fractions that eluted after 55.5 ± 0.1 mL, and showed the cereulide specific signal in the ethanol extracts of the emetic strains, were collected from all strains included in the study and subjected to UPLC-MS/MS [33] to confirm the specificity of the corresponding chromatographic signal for cereulide (see Section 2.3).

In addition, the biological activity of the purified toxin cereulide in the respective positive RPC fractions of the emetic reference strain F4810/72, was tested using human larynx carcinoma (HEp-2) cells, as described previously [4]. The HEp-2-cell assay confirmed the biological activity of cereulide in the respective RPC fraction. Thus, the RPC method described here may be useful, not only for detection of cereulide, but also for the isolation, purification, and concentration of cereulide to be used in further functional in vitro and *in vivo* studies, to fully decipher the mode of action of this toxin.

### 2.3. Method Validation by UPLC-MS/MS-Analysis

The described method for cereulide identification and purification by RPC was cross-validated by means of UPLC-MS/MS. For this purpose, ethanol extracts from each strain of the strain panel were analyzed in parallel by UPLC-MS/MS before and after RPC purification of cereulide. As shown in Table 2, the UPLC-MS/MS analyses confirmed that the peak at 210 nm, after 55.5 mL in RPC, which was uniquely detected in cereulide-producing emetic *B. cereus* strains, indeed contained cereulide. A comparison of the peak areas of cereulide specific peaks in RPC for the different strains and amounts of cereulide determined by UPLC-MS/MS revealed a correlation, which indicates that our RPC method presents a suitable tool for the relative quantification of cereulide and the classification of emetic strains (see Table 2).

All ethanol fractions from emetic strains showing a presumably specific cereulide signal in RPC, were tested positive in the UPLC-MS/MS. Even detection and isolation of cereulide from the low-level emetic *B. cereus* strain RIVM BC379 was possible by RPC. Conversely, no cereulide was detected by UPLC-MS/MS in any of the RPC fractions obtained from the non-emetic *B. cereus* group strains or cereulide-deficient isogenic mutants of the emetic reference strain F4810/72 cereulide, confirming the specificity and sensitivity of the RPC method. Notably, compared to direct analysis of the ethanol extracts by UPLC-MS/MS, the RPC purification protocol resulted in significant enrichment of cereulide (Table 2). Although synthetically produced cereulide has become available in recent years [35], bio-fermentative production of cereulide and subsequent purification using our new RPC method might present a more economical alternative.

Since RPC has been successfully employed to screen for cyanobacterial peptide toxins in water samples [43], it could be assumed that our method might be suitable for screening of cereulide in water or other environmental samples. Although there is increasing evidence that various water reservoirs and water cycles may be important sources of *B. cereus* contamination [44,45,46] and a highly toxic emetic strain isolated from a drinking bottle has been associated with severe cereulide intoxication [34], literally nothing is known about the occurrence of cereulide in water. Thus, our novel RPC method could provide a suitable tool for systematical surveys of water and sediments for cereulide contamination, to decipher the actual contribution of these unexplored niches to cereulide mediated intoxications.

However, it should also be mentioned that the RPC method described here cannot be used for direct detection of cereulide in complex matrices, such as foods, as these complex matrices may interfere with the current approach. Furthermore, in contrast to the SIDA LC-MS/MS for quantitation of cereulide [33], the new RPC does not allow for absolute quantitation of cereulide. Due to the limited resolution of RPC, isotope labelled cereulide, which has been previously shown to be an ideal internal standard for cereulide quantification by UPLC-MS/MS [33], cannot be included in our method. Although valinomycin has been used as surrogate for cereulide in bioassays [5,40], several studies showed that it is not a suitable standard for quantification of cereulide by analytical chemical methods [33,35]. Thus, we have refrained from including it as an internal standard in our RPC method.

Nevertheless, our results from the UPLC-MS/MS analysis of RPC fractions revealed that RPC might be suitable for the relative quantitation of cereulide. For instance, it could be used to classify strains as high- or low-level cereulide producers relative to the emetic reference strain F4810/72, or to test the cereulide production capacity of a mutant strain compared to its parental strain (see Figure 3 and Figure 4). Thus, the RPC method established in this study could become a valuable tool for research, and complementary to UPLC–MS/MS for accurate quantitation of cereulide in outbreak situations and MALDI-ToF MS for rapid detection of emetic *B. cereus* strains in the frame of routine microbial diagnostics.

## 3. Conclusions

In conclusion, RPC was successfully applied for the detection, relative quantification, and isolation of cereulide, which was validated by comparing results with canonical UPLC-MS/MS. Since many microbiological and biochemical research laboratories are equipped with systems for RPC, we expect that our new method can become an economical and easy to implement tool that complements existing more elaborate diagnostic tools for cereulide detection and quantification. In addition, by adjusting the sample preparation protocol and optimizing chromatographic conditions, including adaptations of detector settings and running conditions, the RPC method presented here could also become a suitable tool for cereulide detection in more complex specimens, such as foods, in the future.

## 4. Materials and Methods

### 4.1. Test Set of B. cereus Group Strains

A test set of *B. cereus sensu lato* group strains (*n* = 10) was compiled and used to test the suitability of RPC for cereulide analysis (Table 1). The strain panel included three emetic *B. cereus* strains, one emetic-like strain that shares several physiological features with emetic strains, but is not able to produce cereulide as it lacks the *ces* genes [2,3], as well as three non-emetic *B. cereus* group strains, and three isogenic mutants of the emetic *B. cereus* strain F4810/72, which are either cereulide-deficient or biosynthesize cereulide at different levels.

### 4.2. Cultivation of Bacterial Strains (Step 1)

All bacterial strains were cultivated on LB-Miller (LB) agar plates (10 g tryptone, 5 g yeast extract, and 10 g NaCl per liter) and incubated overnight at 30 °C. Liquid bacterial cultures were inoculated in 3 mL of LB broth, and incubated at 30 °C for 16 to 18 h at 120 rpm. Fresh cultures were inoculated at a final inoculum of 10^3^ CFU/mL in 100 mL of LB broth in bottom-baffled 500-mL-flasks, and incubated at 120 rpm for 24 h at 30 °C, as described previously [8]. Cells were harvested by centrifugation at 8600× *g* for 12 min at room temperature, and the supernatant was discarded. The pellets were frozen in liquid nitrogen and stored at −80 °C until use. Three biological replicates, before and after purification by RPC, were quantified by UPLC-MS/MS analysis.

### 4.3. Cereulide Extraction Procedure (Step 2)

The following extraction procedure was carried out for each culture: After thawing the pellets on ice, 1 g (wet weight) of cell material was transferred to a 50 mL tube. Then, 10 mL of ethanol absolute was added, and the pellet was resuspended gently by shaking or pipetting. Extraction was performed overnight at room temperature on a rocking table. The lid was covered with parafilm to avoid leakage, and was additionally covered with aluminum foil to protect the sample from light.

On the next day, the suspension was centrifuged for 12 min at 8600× g at room temperature and filtered through a 0.2 µm PTFE filter (polytetrafluorethylen; Phenomenex, Aschaffenburg, Germany). The remaining extract was aliquoted to 2 mL and stored at −20 °C until use. The extracts were concentrated 10-fold using a concentrator with an integrated vacuum pump (Eppendorf, Hamburg, Germany) at 45 °C for about 1 h, and extracts from the same strain were pooled to a final volume of 1 mL.

### 4.4. Cereulide Toxin Purification by RPC, (Step 3–4)

The ethanol extract of 1 mL was diluted 1:10 (v/v) in double distilled water. The suspension was gently mixed by inverting the tube 5 times, or until a homogeneously colored suspension was achieved. For RPC, an Äkta™ pure 25 M system with a fraction collector F9-C (GE Healthcare, Solingen, Germany) was employed, and a 5 mL sample loop (PEEK, polyetheretherketone; GE Healthcare, Solingen, Germany) was used for application of the diluted sample extracts.

Due to the highly hydrophobic character of cereulide, the purification of cereulide was performed using a silica based C12 column (Jupiter^®^ 4 µm Proteo 90 Å, LC Column, size 250 * 4.6 mm; Phenomenex, Aschaffenburg, Germany) as RPC, and a pre-column SecurityGuard Cartridge Kit Kj0-4282 (Phenomenex, Aschaffenburg, Germany) with security cartridges filters (Phenomenex, Aschaffenburg, Germany) for the pre-column as the fast protein liquid chromatography (FPLC) system. All solutions were degassed in an ultrasonic bath before use.

UV absorption at 280 nm and 210 nm was simultaneously measured with a UV detector (UV monitor (U9-L), (GE Healthcare, Solingen, Germany). The method-based unit was column volumes (CV), and the default flow rate was set to 0.5 mL/min (control flow to avoid overpressure). PEEK Tubing from the injection valve, column valve, column, UV monitor, and conductivity monitor was changed to PEEK Tubing of ID 0.25 mm OD 1/16” (GE Healthcare, Solingen, Germany) to build-up a higher pressure for cereulide toxin purification.

The running conditions for toxin quantifications were inlet A1 for MQH_2_O, Inlet B1 for ethanol absolute, and inlet B2 for 65% acetonitrile. In this method, all percentage values of ethanol and acetonitrile refer to percentage of volume (% vol). The whole program for cereulide toxin purification and quantification (see Supplementary File S1) included the following steps:

1. Preparation step: the flow rate was set to 0.5 mL/min, and the column was washed with 1 CV of 65% acetonitrile. A linear gradient was performed from 65% to 6.5% of acetonitrile within 2 CV.
2. Equilibration step: the column was equilibrated with 4 CV of 10% ethanol.
3. Sample application: the sample was applied directly to the column using a prefilled 5 mL capillary loop (GE Healthcare, Solingen, Germany).
4. Washing step: an equilibration buffer was used to remove all unbound hydrophilic substances. Fractions of unbound protein were collected using the fraction collector F9-C.
5. Elution step: to elute bound molecules, a 11.50 CV linear gradient of 10% ethanol to ethanol absolute in running buffer (MQH_2_O) was applied. Subsequently, the column was washed with ethanol absolute for 5 CV in running buffer (MQH_2_O). A linear gradient from ethanol absolute to 10% ethanol in running buffer (MQH_2_O) within 3 CV was performed, and the column was washed with 1 CV of 10% ethanol in running buffer (MQH_2_O). Automatic peak fractionation was used to collect fractions >200 mAU (milli-absorbance unit). Cereulide eluting from the column was detected in the 55.5 ± 0.1 mL fraction at a wavelength of 210 nm. Fractions of eluted cereulide were transferred to screw neck vials N9 (1.5 mL, 11.6 × 32 mm with N9 PP screw caps with red rubber; Machery–Nagel, Düren, Germany) for UPLC-MS/MS analysis.
6. Follow-up step 1: the column was washed with 10% ethanol in running buffer (MQH_2_O) for 2 CV.
7. Follow-up step 2: a linear gradient from 10% ethanol to ethanol absolute in running buffer (MQH_2_O) within 1.5 CV was performed.
8. Follow-up step 3: the column was washed with 2 CV ethanol absolute.
9. Equilibration step No. 1: the column was equilibrated with 95% acetonitrile for 4 CV.
10. Equilibration step No. 2: the column was equilibrated with 61.75% acetonitrile for 4 CV.

### 4.5. Method Validation by Ultraperformance Liquid Chromatography-Mass Spectrometry (UPLC-MS/MS)

To confirm the identity of cereulide in the 55.5 mL fraction derived from RPC analysis (see Section 4.3), these fractions were analyzed by UPLC-MS/MS together with the ethanol extracts from the *B. cereus* group strains, which had not been subjected to RPC beforehand.

The mass spectrometric analysis was performed according to the literature on a Waters Xevo TQ-S mass spectrometer (Waters, Manchester, UK) combined with an Acquity UPLC i-class core system (Waters, Milford, MA, USA), comprising a binary solvent manager, sample manager, and column oven [36]. Aliquots (2 µL) of the prepared samples were injected into the UPLC-MS/MS system equipped with a 2.1 × 150 mm, 1.7 µm, UPLC CSH C18 column (Waters, Manchester, UK). The UPLC unit was operated at a flow rate of 0.7 mL/min and a temperature of 55 °C, applying the following gradient with HCOONH_4_ (10 mmol, 0.1% HCOOH) as solvent A, and MeCN (0.25% HCOOH) as solvent B. Chromatography was started at 85% B, increased to 95% B within 8.0 min, increased to 99% B within 0.1 min, kept constant for 0.9 min, decreased to 85% B within 0.1 min, and followed by re-equilibration at 85% B for 0.9 min. Measurements were executed in the positive electrospray ionization (ESI) mode, with quantitative calibration mode consisting of the following ion source parameters: capillary voltage +3.6 kV, sampling cone 50 V, source offset 35 V, source temperature 150 °C, desolvation temperature 650 °C, cone gas 250 L/h, desolvation gas 1100 L/h, collision gas flow 0.15 mL/min, and nebulizer gas flow 7.0 bar. The mass spectrometer was calibrated using a solution of phosphoric acid (0.1% in MeCN) in the range from m/z 40–1963. The UPLC Xevo TQ-S system was operated with MassLynxTM 4.1 SCN 813 Software (Waters, Manchester, UK), and analysis and data processing were completed using TargetLynx (Waters, Manchester, UK). By means of the multiple reaction monitoring (MRM) mode, the ammonium adducts of cereulide (m/z 1170.7 → qualifier: m/z 172.2, 314.2; quantifier: m/z 357.2), and ^13^C_6_-cereulide (m/z 1176.7 → m/z qualifier: 173.2, 316.2; quantifier: m/z 358.2) were analyzed for a duration of 25 ms, observing the mass transitions. ESI + mass and product ion spectra were acquired with direct flow infusion using IntelliStart. The MS/MS parameters were tuned for each individual compound, detecting the fragmentation of the [M + NH_4_] ^+^ molecular ions into specific product ions after collision with argon. All samples were measured in two different dilutions as duplicates. Mean values and standard deviations were calculated from three independent experiments. A detailed protocol of the RPC for the purification of cereulide is provided in the Supplementary File S1.

## Figures and Tables

**Table 1:** *Bacillus cereus* group strains used for method establishment of cereulide purification and quantification by reversed phase chromatography (RPC).

Strain-ID	Relevant Genotype and Characteristics	References
ATCC 14579	Non-emetic *Bacillus cereus* type strain	[38]
F4810/72	Emetic *B. cereus* reference strain, also termed AH187, isolated from vomit; emetic food-borne outbreak in UK	[3,26]
ATCC 10792	*Bacillus thuringiensis* type strain	[39]
RIVM BC90 ^a^	*B. cereus* isolated from human faces; diarrheal outbreak in The Netherlands	[3,26]
F48*∆cesP/polar* ^b^	F4810/72 ∆*cesP*::*spc*, Spc^r^; cereulide deficient due to transcriptional inactivation of *cesABCD* genes	[9]
F48*∆abrB* ^c^	F4810/72 ∆*abrB*::*spc*, Spc^r^; cereulide overproduction due to deletion of transcription regulator *abrB*	[40]
F48*∆pBCE* ^b^	F4810/72 ∆*pBCE270;* cereulide deficient due to deletion of *ces* locus encoding plasmid pBCE270	[Dommel and Ehling-Schulz; unpublished]
F5881/94	Emetic toxin producing *B. cereus* strain isolated from Chinese takeaway fried rice; emetic food-borne outbreak in UK	[3,26]
RIVM BC379	Emetic toxin producing *B. cereus* isolated from chicken; The Netherlands	[34]
*Bacillus anthracis* Sterne	Attenuated vaccine strain, which lacks virulence plasmid pXO2	[41]

^a^ emetic-like strain as defined by Ehling-Schulz et al. [3]; ^b^ cereulide deficient mutant; ^c^ cereulide overproducing mutant.

**Table 2:** Quantification of cereulide by means of UPLC-MS/MS before/after cereulide purification/enrichment by RPC, and comparison of cereulide-specific peak areas at 55.5 mL. Part of the ethanol extracts from each strain was subjected directly to LC-MS/MS analysis, while parts of the extracts were processed via RPC. The respective RPC fraction eluting after 55.5 ± 0.1 mL was subsequently analyzed by UPLC-MS/MS. The cereulide-specific peak areas at 55.5 mL were quantified for comparison of the relative amounts of UPLC-MS/MS- and RPC-enriched cereulide fractions. Abbreviations: N.D.: not detected. Std. Dev.: standard deviation ^2^. mAU: milli-absorbance unit.

	Ethanol Extracts of *B. cereus* Directly Subjected to UPLC-MS/MS Analysis	RPC-Enriched Cereulide Fractions (55.5 mL) Subjected to UPLC-MS/MS	Cereulide-Specific Peak Area of RPC at 55.5 mL
Strain-ID ^1^	Cereulide [µg/mL] ± Std. Dev. ^2^	Cereulide [µg/mL] ± Std. Dev. ^2^	mL*mAU ± Std. Dev. ^2^
**F4810/72**	3.8 ± 2.7	33.4 ± 10.0	91.6 ± 28.4
**F48*∆abrB***	10.4 ± 1.3	82.9 ± 18.1	113.8 ± 27.0
**F5881/94**	17.9 ± 7.2	95.7 ± 12. 9	127.8 ± 26.4
**RIVM BC379**	2.1 ± 1.3	24.4 ± 6.4	23.0 ± 10.7
ATCC 14579	N.D.	N.D.	N.D.
RIVM BC90	N.D.	N.D.	N.D.
F48*∆cesP/*polar	N.D.	N.D.	N.D.
F48*∆pBCE*	N.D.	N.D.	N.D.
ATCC 10792	N.D.	N.D.	N.D.
*B. anthracis* Sterne	N.D.	N.D.	N.D.

^1^ Emetic strains producing cereulide are indicated in bold. ^2^ Means and standard deviations are derived from *n* = 3 independent experiments (for details see Section 4).

## Data Availability

Data is contained within the article or Supplementary Materials.

## Supplementary Materials

The following are available online at https://www.mdpi.com/2072-6651/13/2/115/s1, File S1: Full Method Protocol.
