# Supplementary material for: Detection and Isolation of Emetic Bacillus cereus Toxin Cereulide by Reversed Phase Chromatography
Source: Toxins (Basel). 2021 Feb 4;13(2):115. doi: 10.3390/toxins13020115 (PMC7915282; doi:10.3390/toxins13020115)
Supplement: Supplementary file 1 [file toxins-13-00115-s001.pdf]

Supplementary Materials: Detection and Isolation of Emetic *Bacillus cereus* Toxin Cereulide by Reversed Phase Chromatography

Eva Maria Kalbhenn, Tobias Bauer, Timo D. Stark, Mandy Knüpfer, Gregor Grass and Monika Ehling-Schulz

File S1: Full Method Protocol

Step 1: Cultivation of bacterial strains

Materials:

- LB-Miller (LB) broth (10 g tryptone, 5 g yeast extract and 10 g NaCl per liter) and LB agar plates
- Bottom-baffled flasks (volume of 500 mL)
- Orbital shaker with rotation and temperature control
- Liquid nitrogen

Procedure:

1. Strike bacterial strains on LB agar plates. Incubate plates overnight at 30 °C.
2. Incubate bacterial pre-cultures in 3 mL of LB broth at 30 °C for 16 to 18 h at 120 rpm.
3. Inoculate fresh cultures with a final inoculum of 10^3 CFU/mL in 100 mL of LB broth and incubate cultures at 120 rpm for 24 h at 30 °C, as described previously [1].
4. Harvest cells by centrifugation at 8600 × g for 12 min at room temperature and discard supernatant.
5. Freeze pellets in liquid nitrogen for storage at −80 °C.

Step 2: Extraction procedure

Materials:

- Precision scale
- Ethanol absolute
- Rocking table platform
- Parafilm strips
- Aluminum foil
- Syringes (volume of 10 mL)
- PTFE (Polytetrafluorethylen) filters with a pore size of 0.2 µm (Phenomenex,Aschaffenburg, Germany)
- SpeedVac (concentrator with integrated vacuum pump, Eppendorf)

Procedure:

1. Thaw pellets on ice.
2. For each strain, weigh 1 g (wet weight) of cell material into a 50 mL tube.
3. Add 10 mL of ethanol absolute and resuspend the pellet by gently shaking or pipetting.
4. Extract overnight at room temperature on a rocking table. The screw cap should be covered by parafilm to avoid leakage. The samples should also be covered with aluminum foil to protect them from light.
5. On the next day, centrifuge the suspension for 12 min at 8600 × g at room temperature.
6. Filtrate the extracts to remove any remaining cell debris by using a syringe and PTFE filters (pore size of 0.2 µm). Aliquot the extracts to 2 mL for each strain and store them at −20 °C until use.
7. Concentrate extracts to 200 µL using a concentrator at 45 °C for about 1 h. Pool extracts from each strain into a single tube with a final volume of 1 mL.

Step 3: Cereulide toxin purification by reversed phase chromatography (RPC)

Materials:

- Äkta™ pure 25M (GE Healthcare, Solingen, Germany) with UV monitor U9-M for triple wavelengths detection
- Fraction collector F9-C for collection in various tube types (GE Healthcare)
- UNICORN 7 control software (GE Healthcare, Solingen, Germany)
- 5 mL sample loop, PEEK (Polyetheretherketone), (GE Healthcare)
- Acetonitrile
- Ethanol absolute
- Inject™ syringe 5 mL luer lock (Braun)
- Union luer female-17/Male (GE Healthcare)
- PEEK Tubing, 2 m ID 0.25 mm OD 1/16" (GE Healthcare)
- PEEK Tubing, 2 m, ID 0.75 mm, OD 1/16" (GE Healthcare)
- Pre-column: Security Guard Cartridge Kit KJ0-4282 (Phenomenex) and Security Guard Cartridges (Filters for pre-column), C12 4 × 3.0 mm ID (Phenomenex)
- Jupiter® 4 µm Proteo 90 Å, LC Column Size 250*4.6 mm (Phenomenex)
- Screw neck vials N9, 1.5 mL, 11.6 × 32 mm with N9 PP screw caps with red rubber (Machery –Nagel)

Note: Prepare all solutions in advance. All solutions should be degassed in an ultrasonic bath before use. Make sure the Äkta™ pure 25M system is set-up according to the manufacturer's instructions (GE Healthcare). In this method, all percentage values of ethanol and acetonitrile refer to percentage of volume (% vol).

Procedure:

- Dilute ethanol extract 1:10 (v/v) in MQH_2O. Gently mix by inverting the tube 5 times, or until a homogeneously colored suspension is achieved.
- Pre-fill the 5 mL sample loop of the Äkta™ pure 25M system with 5 mL of the sample.
- Due to the highly hydrophobic character of cereulide, for the purification of cereulide a silica based C12 column (Jupiter® 4 µm Proteo 90 Å, LC Column, size 250 * 4.6 mm (Phenomenex)) as reversed phase chromatography (RPC) and a pre-column SecurityGuard Cartridge (Phenomenex) as fast protein liquid chromatography (FPLC) system is recommended.

Method Settings:

- Reverse phase chromatography (RPC)
- Inlet A1 and inlet B1. Unless stated otherwise, use the same inlets as mentioned in the method settings.
- UV absorption is simultaneously measured with the UV detector (UV monitor (U9-L), (GE Healthcare)) at 280 nm and 210 nm.
- Air sensor enables the air sensor alarm for the built-in air sensor at inlet A and B.
- Method based unit is column volumes (CV).
- To minimize gas formation in the system, use a flow restrictor.
- Set the flow rate to 0.5 mL/min (control the flow to avoid overpressure). Use always the same flow rate as mentioned in the method settings, if not stated otherwise.
- Column position is variable.
- Fractionation collector is only required for elution.

Note: In general PEEK tubing is ID 0.75 mm, OD 1/16" (GE Healthcare). Change PEEK tubing from injection valve, column valve, column, UV monitor, and conductivity monitor to PEEK tubing of ID 0.25 mm OD 1/16" (GE Healthcare) to build-up a higher pressure for cereulide purification.

Running conditions for toxin quantification:

- Inlet A1: MQH_2O.
- Inlet B1: Ethanol absolute
- Inlet B2: 65% acetonitrile

Program for Äkta™ pure 25M:

- Preparation step:
    - Set flow rate to 0.5 mL/min and wash the column with 1 CV of 65% acetonitrile. Perform a linear gradient from 65% to 6.5% of acetonitrile within 2 CV.
- Equilibration step:
    - Reseting of the UV monitor is recommended if the equilibration occurs before purification.
    - Equilibrate column with 4 CV of 10% ethanol.
- Sample application:
    - Apply sample directly to the column using the prefilled 5 mL capillary loop.
- Washing step:
    - Use equilibration buffer to remove all unbound hydrophilic substances. Fractions of unbound protein were collected using the fraction collector F9-C.
- Elution step:
    - To elute bound molecules, an 11.50 CV linear gradient from 10% ethanol to ethanol absolute in running buffer (MQH_2O) is applied. Subsequently, the column is washed with ethanol absolute for 5 CV in running buffer (MQH_2O). Perform a linear gradient from ethanol absolute to 10% ethanol in running buffer (MQH_2O) within 3 CV, and wash the column with 1 CV of 10% ethanol in running buffer (MQH_2O). Fractions with peaks >200 mAU are automatically collected using a fraction collector F9-C (GE Healthcare).
    - Cereulide eluting after 55.5 ±0.1 mL from the column is detected at a wavelength of 210 nm, and collected by automatic "peak fractionation" >200 mAU.
    - Fractions of eluted cereulide are transferred to screw neck vials for UPLC–MS/MS analysis.
- Follow-up step 1:
    - Wash column with 10% ethanol in running buffer (MQH_2O) for 2 CV.
- Follow-up step 2:
    - Perform a linear gradient from 10% ethanol to ethanol absolute in running buffer (MQH_2O) within 1.5 CV.
- Follow-up step 2:
    - Wash column with ethanol absolute for 2 CV.

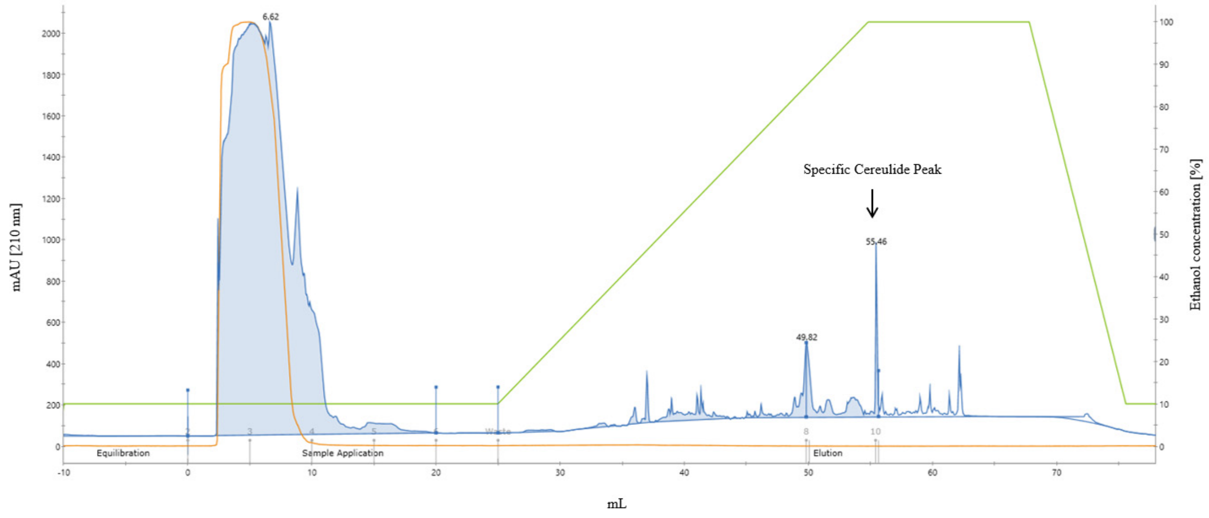

Figure 1: Reversed phase chromatogram of ethanol extracts from *B. cereus* F4810/72 wild-type (blue line) for detection and isolation of cereulide. The strain was grown in LB broth for 24 h at 30 °C, 120 rpm, cereulide extracted from cell pellet with ethanol absolute over-night and 10-fold concentrated. Ethanol extracts were 1:10 diluted in water (v/v) and analyzed by RPC using a silica based C12 column (Jupiter Proteo 250 × 4.6 mm, particle Size 4 µm, pore Size 90Ǻ; Phenomenex, Germany) on an Äkta™ pure 25M system. The cereulide containing peak at 55.5 mL was collected and quantified by LC-MS/MS. The different steps of the RPC (equilibration, sample application, elution) are indicated on the orange line. Ethanol gradient is shown in green. Fractions collected are labelled in grey and indicated by numbers.

<u>*Running conditions for column regeneration and storage:*</u>

- Inlet A1: MQH_2O
- Inlet B2: 95% acetonitrile in MQH_2O

<u>*Program for Äkta™ pure 25M:*</u>

- Equilibration step No. 1:
    - Reseting of the UV monitor is recommended, if the equilibration occurs before purification.
    - Equilibrate the column with 95% acetonitrile for 4 CV.
- Equilibration step No. 2:
    - Reseting of the UV monitor is recommended, if the equilibration occurs before purification.
    - Equilibrate the column with 61.75% acetonitrile for 4 CV.

References:

1. Dommel, M.K.; Lücking, G.; Scherer, S.; Ehling-Schulz, M. Transcriptional kinetic analyses of cereulide synthetase genes with respect to growth, sporulation and emetic toxin production in *Bacillus cereus*. *Food Microbiol.* **2011**, *28*, 284–290.
